# Sociodemographic and clinical characteristics of 1,234 individuals diagnosed with trichotillomania in the Swedish National Patient Register

**DOI:** 10.1038/s41598-025-95416-w

**Published:** 2025-03-26

**Authors:** Luis C. Farhat, Kayoko Isomura, Lorena Fernández de la Cruz, Anna Sidorchuk, Ralf Kuja-Halkola, Isabell Brikell, Zheng Chang, Brian M. D’Onofrio, Henrik Larsson, Paul Lichtenstein, David Mataix-Cols

**Affiliations:** 1 Department of Psychiatry, Faculdade de Medicina FMUSP, Universidade de São Paulo, São Paulo, Brazil; 2 Centre for Psychiatry Research, Department of Clinical Neuroscience, Child and Adolescent Psychiatry Research Centre, Karolinska Institutet & Stockholm Health Care Services, Gävlegatan 22B, 8th floor, 113 30 Region Stockholm, Sweden; 3 Department of Medical Epidemiology and Biostatics, Karolinska Institutet, Stockholm, Sweden; 4 Department of Global Public Health and Primary Care, University of Bergen, Bergen, Norway; 5 Department of Biomedicine, Aarhus University, Aarhus, Denmark; 6 Department of Psychological and Brain Sciences, Indiana University, Bloomington, IN US; 7 School of Medical Sciences, Örebro Universitet, Örebro, Sweden; 8 Department of Clinical Sciences, Lunds Universitet, Lund, Sweden

**Keywords:** Hair-pulling disorder, Body-focused repetitive behaviors, Obsessive–Compulsive disorder, Disruptive, Impulse Control, and Conduct Disorders, Population registers, Epidemiology, Outcomes research

## Abstract

Trichotillomania is an understudied, underrecognized, and difficult-to-treat psychiatric disorder that affects about 1–2% of the population, predominantly women. This study aimed to characterize the sociodemographic and clinical characteristics of a Swedish cohort of individuals with a diagnosis of trichotillomania assigned in specialist services across the country. Through the Swedish National Patient Register, we identified all individuals with an ICD-10 diagnosis of trichotillomania between January 1^st^, 1997 and December 31^st^, 2020. The cohort included 1,234 individuals, with a median age at first diagnosis of 25 years (interquartile range, 16–35). Most individuals were female (85%) and single (82%). Most (79%) individuals had a comorbid psychiatric disorder diagnosed during the study period. Anxiety-related disorders (65%), depressive disorders (48%), and neurodevelopmental disorders (39%) were the most frequent co-occurring diagnoses. Most (72%) individuals were dispensed psychotropic medications during the period ranging from 12 months prior to and 12 months after the first trichotillomania diagnosis. Antidepressants (58%), hypnotics/sedatives (36%), and anxiolytics (31%) were the most frequently dispensed medications. Altogether, the characteristics of individuals diagnosed with trichotillomania in Swedish specialist services were similar to those reported in previous literature, opening the possibility of register-based research into this underdiagnosed and understudied condition.

## Introduction

Trichotillomania, also known as hair-pulling disorder, is a psychiatric disorder characterized by poor control of, and sustained engagement in, hair pulling of one’s own hair, leading to hair loss and related distress and/or interference^1,2^. A recent systematic review and meta-analysis of population-based surveys estimated the average lifetime prevalence of the disorder to be between 1 and 2% of the general population, and likely slightly higher in women compared to men^3^. Those who live with this condition may experience impairments in social and academic or occupational functioning, as well as lower quality of life^4,5^. Unfortunately, trichotillomania remains surprisingly difficult to treat. Psychotherapeutic interventions have shown to be efficacious against waitlist in a few small randomized controlled trials, but the confidence around their effects is low and there are barriers to their effective implementation in the community^4^. There are no Food and Drug Administration-approved medications for hair-pulling symptoms^6^.

Trichotillomania is considerably understudied, compared to other psychiatric disorders, including related conditions such as obsessive–compulsive disorder. To date, most studies of trichotillomania have been based on small samples recruited from research-active clinics^7–11^or Internet-based surveys^4,12^. Individuals with trichotillomania who respond to advertisements for participation in research studies may not be representative of the pool of individuals with trichotillomania who seek help for their hair-pulling behaviors. Therefore, it is unclear whether the findings from those studies are generalizable to the greater population of treatment-seeking individuals with trichotillomania. Studies with increased sample sizes and wider coverage of recruitment are needed in the field because the generalizability of findings from single-site recruitment efforts to the larger community remains a concern.

Population-based registers from Nordic countries (e.g., Sweden and Denmark) have been leveraged to identify and characterize nationwide cohorts of individuals with various psychiatric disorders^13^ but, to our knowledge, trichotillomania has not been the focus of register-based research. In this study, we sought to identify a Swedish nationwide cohort of individuals diagnosed with trichotillomania in specialist services through the National Patient Register (NPR) and describe their sociodemographic and clinical (i.e., psychiatric comorbidities and dispensed psychotropic medications) characteristics. We predicted that the sociodemographic and clinical characteristics of this nationwide cohort from Sweden would resemble those of individuals with trichotillomania from international samples previously described in the literature.

## Methods

The study was approved by the Swedish Ethical Review Authority (reference number: 2020–06540). Informed consent was not required because the study was based on the registers and the included individuals were not identifiable at any time. All procedures were performed in accordance with the relevant guidelines and regulations and the Declaration of Helsinki.

### Data Sources

We used the Swedish personal identity number^14^, a unique identification number allocated to all Swedish residents, to link several Swedish population-based registers. Diagnostic information on trichotillomania and other psychiatric disorders was retrieved from the NPR^15^, which comprises clinical records from all inpatient care (complete nation coverage since 1987) and outpatient specialist services (from 2001) in Sweden. In the NPR, diagnoses are recorded based on the International Classification of Diseases (ICD), eighth (1973–1986), ninth (1987–1996), and tenth (1997 onward) editions. Clinical data on intellectual disability were supplemented by information from the Halmstad University Register on Pupils with Intellectual Disability (HURPID)^16^, which is a national database of people who graduated from an upper secondary school for students with intellectual disability between January 1^st^, 2001 and December 31^st^, 2011. Sociodemographic characteristics were extracted from the Total Population Register^17^, which contains demographic data on all Swedish inhabitants since 1968, and the Longitudinal Integration Database for Health Insurance and Labour Studies^18^, which annually integrates data on income, labor market, education, and social sectors from all Swedish residents since 1990. Data on psychotropic medications were acquired from the Prescribed Drug Register (PDR)^19^ that comprises information on prescription medication dispensed across all pharmacies in Sweden since July 2005 onwards, registered according to the Anatomical Therapeutic Chemical (ATC) Classification System codes.

### Participants

The source population included all individuals living in Sweden between January 1^st^, 1997 (implementation of the ICD-10 codes in Sweden) and December 31^st^, 2020 (end of the study period) for at least one uninterrupted year (to equalize a probability of having health and administrative data recorded in the registers). From them, we constructed a study cohort by selecting all individuals with at least one inpatient or specialist outpatient diagnosis of trichotillomania (ICD-10 code F63.3), recorded in the NPR between 1997 and 2020. Cases of trichotillomania were identified irrespective of whether they were primary or secondary diagnoses.

Individuals were excluded from the cohort if they had a diagnosis of intellectual disability in the NPR (ICD-10 codes F70-F79) or a record of completing an upper secondary school for pupils with intellectual disability, according to the HURPID. Stereotypic movement disorders are common in individuals with intellectual disabilities and share phenomenological characteristics with body-focused repetitive behavior disorders such as trichotillomania^20^. These similarities could contribute to misdiagnosis in routine clinical practice. There is ongoing debate regarding the extent to which stereotypic movement disorders and trichotillomania should be conceptualized as distinct psychiatric conditions^20^. However, there is also agreement that stereotypic movements in the context of an intellectual disability and in the context of trichotillomania are distinct, with scant evidence on the overlap for these conditions. Hence, we judged that the exclusion of participants with intellectual disability was conservatively appropriate. Individuals with conflicting information in the NPR (e.g., died before the date of the first trichotillomania diagnosis) were also excluded.

For the analysis of psychotropic medication use, a subcohort of individuals with a diagnosis of trichotillomania was created by excluding those who died or emigrated the country before July 1, 2005, when the PDR was introduced.

### Sociodemographic characteristics, psychiatric comorbidities, and psychotropic medications

For each individual with a diagnosis of trichotillomania, we retrieved sociodemographic data, including sex (female or male), country of birth (Sweden or abroad), level of education (elementary [≤ 9 years], secondary [10–12 years], or higher education [> 12 years]), civil status (single, married/cohabiting, or divorced/widowed), and family disposable income level (lowest 20%, middle 60%, or top 20%). For education, civil status, and income, we used the information available at the time of the first trichotillomania diagnosis, or the nearest year available.

Furthermore, data on comorbid psychiatric disorders diagnosed in specialist services were collected in two ways: 1) if diagnosed from 1997 and until or on the same date of the first trichotillomania diagnosis and 2) if diagnosed at any time during the study period (1997–2020), before or after the date of the trichotillomania diagnosis. Psychiatric disorders were grouped into: neurodevelopmental disorders (NDDs), including attention-deficit/hyperactivity disorder (ADHD), pervasive developmental disorders, and Tourette syndrome or chronic tic disorders; schizophrenia or other psychotic disorders; bipolar disorders; depressive disorders; anxiety-related disorders, including anxiety and phobic disorders, obsessive–compulsive disorder, and reaction to severe stress and adjustment disorders; eating disorders; and emotionally unstable personality disorder (see ICD codes in Supplementary Table 1).

For a sub-cohort of individuals with a diagnosis of trichotillomania who were alive and living in Sweden ever between July 1, 2005 and December 31, 2020, we collected data on psychotropic medications dispensed within a period from 12 months prior to and 12 months after the first trichotillomania diagnosis. From the PDR, dispensation records were retrieved for the following psychotropic medications: ADHD medications (including methylphenidate and other stimulants or nonstimulants), antidepressants (including selective serotonin reuptake inhibitors [SSRIs], serotonin-noradrenaline reuptake inhibitors [SNRIs], clomipramine, and other antidepressants), anxiolytics (including benzodiazepine and non-benzodiazepine anxiolytics), hypnotics (including benzodiazepine and non-benzodiazepine hypnotics), antipsychotics (including typical and atypical), lithium, antiepileptics (including lamotrigine, divalproex, and other antiepileptics), antiparkinsonian medications, naltrexone, analgesics (including opioids, other analgesics and antipyretics, and antimigraine preparations), and other nervous system medications (see ATC codes in Supplementary Table 2).

### Statistical analyses

To descriptively characterize individuals with trichotillomania, we reported the distribution (i.e., counts and corresponding percentages for categorical variables; median and interquartile range [IQR] and/or mean and standard deviation [SD] for continuous variables) of sociodemographic characteristics, psychiatric comorbidities, and dispensed psychotropic medications. Distribution of the number of new trichotillomania diagnoses recorded in each year throughout the study period (1997–2020) and in different age groups was visualized in graphs. Data management and analyses were performed using SAS, version 9.4 (SAS Institute Inc.).

## Results

### Study cohort

**Fig. 1:**
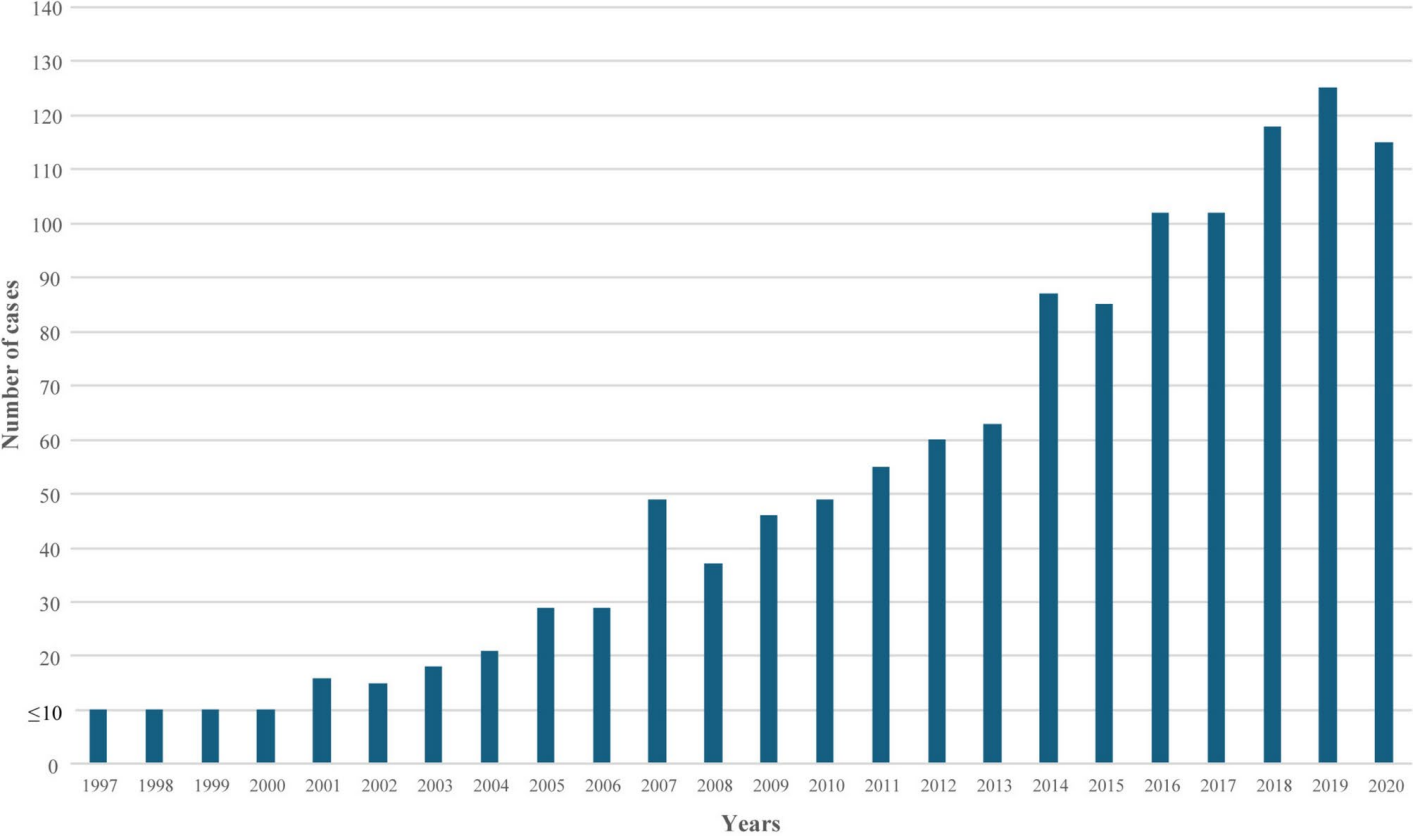
Trichotillomania diagnoses in the Swedish National Patient Register. The figure illustrates the distribution of new trichotillomania diagnoses recorded in the Swedish National Patient Register for each year throughout the study period (1997 to 2020). In the y-axis, ≤ 10 indicates that 10 or fewer diagnoses were made in the corresponding year.

Out of 12,987,753 individuals who lived in Sweden during 1997–2020 for at least one uninterrupted year, 1,290 had a diagnosis of trichotillomania recorded in the NPR. Of those, 56 (4.34% of those with a diagnosis of trichotillomania) individuals were excluded due to having a diagnosis of intellectual disability, leaving 1,234 individuals with trichotillomania in the study cohort (0.01% of the source population). As shown in Fig. 1, there was a steady increase in the number of new trichotillomania diagnoses throughout the study period, particularly from the year 2001, when the diagnoses from outpatient specialist services were included in the NPR.

### Sociodemographic characteristics

**Fig. 2:**
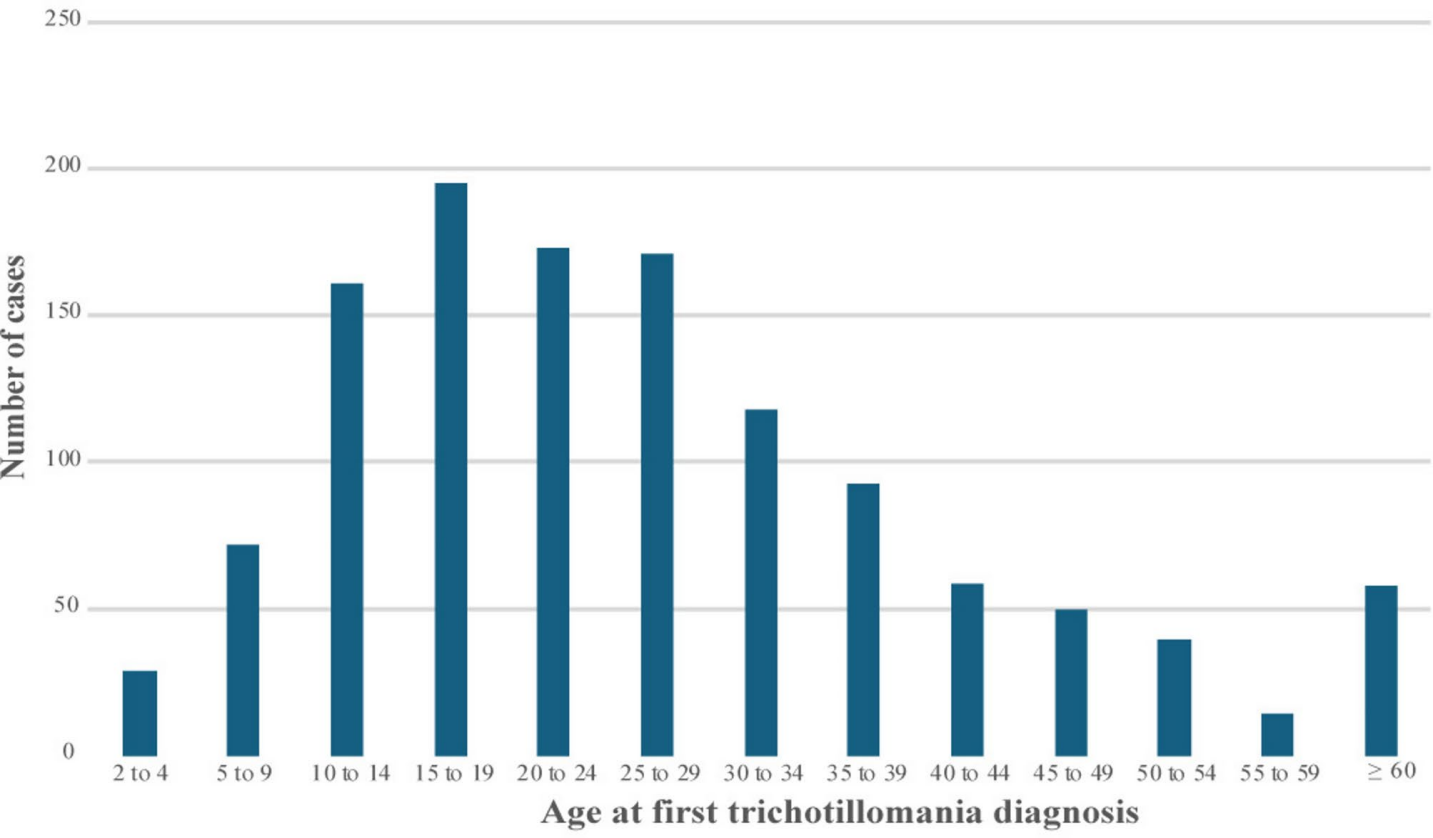
Age at first diagnosis of trichotillomania. The figure illustrates the distribution of trichotillomania diagnoses recorded in the Swedish National Patient Register in 1997–2020 among 1,234 study participants according to the age at first diagnosis.

The median age at first diagnosis of the 1,234 individuals with a diagnosis of trichotillomania in the cohort was 24.60 years ([IQR, 16.35–35.30]; mean age, 27.60 years, [SD, 15.79]). In most cases, individuals with a diagnosis of trichotillomania received their first diagnosis in adolescence and early-to-mid adulthood, with fewer incident diagnoses recorded in other age groups, particular among the elderly (Fig. 2).

Individuals with trichotillomania were predominantly female (n = 1,047, 85%), born in Sweden (n = 1,034, 84%), and single (n = 1,009, 82%). Furthermore, by the time when trichotillomania was first diagnosed, over one third of cohort members had elementary education (n = 450, 36%) as the highest attained educational level, and more than half had a middle level of disposable family income (n = 660, 53%) (Table 1).

**Table 1:** Distribution of sociodemographic characteristics among 1,234 individuals with trichotillomania diagnoses recorded in the Swedish National Patient Register in 1997–2020.

Sociodemographic characteristics	Individuals with trichotillomania	
	**N**	**%**
**Sex**		
Female	1,047	85
Male	187	15
**Birth year**		
1969 or earlier	190	15
1970 to 1979	157	13
1980 to 1989	270	22
1990 to 1999	345	28
2000 to 2009	248	2
2010 or later	27	2
**Country of birth**		
Sweden	1,034	84
Abroad	200	16
**Educational level attained**^**a**^		
Elementary education	450	36
Secondary education	348	28
Higher education	310	25
Missing	126	10
**Marital status**^**a**^		
Single	1009	82
Married or cohabiting	136	11
Divorced or widowed	0	0
Missing	89	7
**Family disposable income level**^**a**^		
Lowest 20%	213	17
Middle 60%	660	53
Top 20%	272	22
Missing	89	7

^a^Values correspond to the year of trichotillomania diagnosis or the nearest year available.

### Psychiatric comorbidities

Table 2 presents the distribution of comorbid psychiatric diagnoses recorded by specialist physicians amongst individuals with a diagnosis of trichotillomania. A total of 841 (68%) individuals had at least one other psychiatric disorder recorded before or on the same date as the first diagnosis of trichotillomania, with anxiety-related disorders (n = 630, 51%), depressive disorders (n = 441, 36%), and NDDs (n = 298, 24%) being the most frequent co-occurring diagnoses. When considering the entire study period (both *before* and *after* the first trichotillomania diagnosis), as many as 980 (79%) individuals had a recorded comorbid psychiatric disorder. Similarly, anxiety-related disorders (n = 493, 65%), depressive disorders (n = 363, 48%), and NDDs (n = 246, 39%) were the most frequent comorbidities. Additionally, approximately 7% and 8% of individuals had a diagnosis of emotionally unstable personality disorder and eating disorders, respectively (10% during the whole study period for each comorbidity).

**Table 2:** Comorbid psychiatric disorders recorded in the Swedish National Patient Register among 1,234 individuals with trichotillomania.

	**Comorbid psychiatric diagnoses**			
	**Diagnosed prior to or on the day of the first diagnosis of trichotillomania**		**Diagnosed at any time during the study period**	
	**N**	**%**	**N**	**%**
**Any psychiatric disorder**	841	68	980	79
**Neurodevelopmental disorders**	298	24	480	39
Attention-deficit/hyperactivity disorder	234	19	401	34
Pervasive developmental disorders	114	9	195	16
Tourette syndrome or chronic tic disorders	32	3	32	3
**Schizophrenia and other psychotic disorders**	40	3	64	5
**Bipolar disorders**	55	4	98	8
**Depressive disorders**	441	36	588	48
**Anxiety-related disorders**	630	51	803	65
Anxiety and phobic disorders	478	39	659	53
Obsessive–compulsive disorder	219	18	297	24
Reaction to severe stress and adjustment disorders	192	16	334	27
**Eating disorders**	99	8	129	10
**Emotionally unstable personality disorder**	81	7	128	10

### Dispensed psychotropic medications

A total of 1,228 individuals with a diagnosis of trichotillomania were alive and living in the country at the time when the PDR started (corresponding to 99.5% of the total trichotillomania cohort). For this sub-cohort, Table 3 presents the distribution of psychotropic medications dispensed within a period from 12 months before to 12 months after the first trichotillomania diagnosis. As many as 888 (72%) sub-cohort members had at least one record of any psychotropic medication dispensed in proximity to the diagnosis date. Antidepressants (n = 715, 58%), hypnotics/sedatives (n = 447, 36%), and anxiolytics (n = 385, 31%) were the most frequently dispensed types of medications.

**Table 3:** Psychotropic medications dispensed during the period from 12 months before to 12 months after the first diagnosis in a sub-cohort of 1,228 individuals with trichotillomania^a^.

	**Individuals with trichotillomania (N = 1,228)**	
	**N**	**%**
**Any psychotropic medication**	888	72
**ADHD medications**	229	19
Methylphenidate	176	14
Other ADHD medications	124	10
**Antidepressants**	715	58
SSRIs	574	47
SNRIs	159	13
Clomipramine	38	3
Other antidepressants	227	18
**Anxiolytics**	385	31
Benzodiazepine anxiolytics	176	14
Non-benzodiazepine anxiolytics	271	22
**Hypnotics/sedatives**	447	36
Benzodiazepine hypnotics	249	20
Non-benzodiazepine hypnotics	317	26
**Antipsychotics**	219	18
Typical antipsychotics	45	4
Atypical antipsychotics	195	16
**Lithium**	16	1
**Antiepileptics**	111	9
Lamotrigine	82	7
Divalproex	13	1
Other antiepileptics	28	2
**Antiparkinsonian medications**	11	1
**Naltrexone**	15	1
**Analgesics**	265	22
Opioids	139	11
Other analgesics and antipyretics	197	16
Antimigraine preparations	41	3
**Other nervous system medications**	21	2

^a^The subcohort contains individuals with trichotillomania diagnosis who did not die or emigrated from Sweden before July 1, 2005, when the Prescribed Drug Register was introduced.

*Abbreviations:* ADHD, attention-deficit/hyperactivity disorder; SNRIs, serotonin-noradrenaline reuptake inhibitors; SSRI, selective serotonin reuptake inhibitors.

## Discussion

To our knowledge, this was the first nationwide register-based study of trichotillomania conducted anywhere. We extracted data from the Swedish population-based registers to identify a nationwide cohort of 1,234 individuals diagnosed with trichotillomania within specialist services. We described those individuals regarding sociodemographic characteristics, psychiatric comorbidities, and psychotropic medications dispensed in proximity to the date of the first trichotillomania diagnosis.

Our data indicate that the diagnoses of trichotillomania made in specialist care likely represent a very small proportion of all possible individuals with trichotillomania in Sweden, if we consider that the expected prevalence in the population is around 1–2%^3^. However, most population-based surveys of trichotillomania have determined the presence, or absence, of the disorder considering self-reported answers to questionnaires querying diagnostic criteria. Because trichotillomania cannot be reliably diagnosed without rigorous clinical assessments by trained professionals, it is possible that the prevalence estimates of 1–2% may be overestimated. Additionally, naturally, not all individuals with the disorder may seek help for their condition, and the rates of help-seeking among individuals with trichotillomania are unclear. Nevertheless, the proportion of individuals with trichotillomania in our study, approximately 0.01% of the general population, is striking. When we plotted the yearly number of new diagnoses of trichotillomania, we observed a clear upward trend, varying from two trichotillomania records in 1997 to 115 in 2020, with the five last years of the study period recording over 100 new cases per year, which may still be a small number for a country of approximately 10 million people. Additional nationwide register-based data from specialist services in other countries would be informative to learn whether this pattern is specific to Sweden or a general trend. In the meantime, it is sensible to assume that trichotillomania may also be underdiagnosed in other countries. Individuals with trichotillomania are often ashamed of their hair-pulling symptoms and feel like their health providers know little of their condition^4,21^, which may contribute to underreporting and underdiagnosis. Besides, individuals with trichotillomania may be more likely to seek help from counselors, social workers or primary care physicians^4^, and may not receive care in secondary or tertiary specialist services. While more research is needed to definitively determine whether there is a larger population of individuals needing help for hair-pulling behaviors and urges, in the meantime more could be done to raise awareness and create care pathways for these individuals in Sweden and other countries.

We found that most (85%) individuals with a diagnosis of trichotillomania were female, which aligns with previous descriptions of participants recruited for phenomenological, brain imaging, neurocognitive, and treatment studies of trichotillomania^4,7,22,23^. In contrast, evidence from epidemiological studies indicates a more balanced sex distribution of trichotillomania in the general population^3^. Due to sociocultural norms, females may be more likely to notice hair loss and experience hair-pulling-related distress and impairment, which in turn may result in higher likelihood of seeking help, leading to an increased number of diagnoses for this group in clinical settings.

Our findings also indicated that, while trichotillomania was rarely diagnosed in young children (ages 2–9 years), half of the individuals with the condition had their first diagnosis by the age of 25 years, and the majority (75%) had a diagnosis by the age 35 years. Previous research indicates that trichotillomania severity may peak around the transition from adolescence onto adulthood^10^. It has been reported that most individuals with trichotillomania had their onset during adolescence (mean age 12 years old), with a smaller group of individuals having a mid-adulthood onset (mean age 36 years)^24^. Our results, showing a later age of first diagnosis, most likely respond to the known delays in seeking specialist help^4^and receiving a diagnosis^4,21^and align with previous reports on help-seeking samples^22^.

With regards to civil status, our findings align with those from the Trichotillomania Impact Project (TIP), which indicated that most individuals with trichotillomania who participated in an Internet survey were single^4^. Hair-pulling symptoms may affect self-esteem (e.g., individuals with trichotillomania may feel unattractive) and interfere with interpersonal relationships^25^. However, our findings could be explained, at least partly, by comorbid psychiatric disorders, considering the observed high comorbidity rates. Alternatively, because we considered civil status for the year of the trichotillomania diagnosis, or the closest year available, it is possible that our findings on civil status mainly reflected the relatively young age of some individuals in our cohort when they first received a diagnosis of trichotillomania (e.g., about half of them were younger than 30 years at the time of the diagnosis).

A majority (68%) of individuals with trichotillomania had at least one comorbid psychiatric disorder recorded prior to or at the same time as the first diagnosis of trichotillomania, and this proportion was higher (79%) when considering the comorbidities diagnosed during the whole study period. These figures are generally in line with reports comprising hundreds of individuals with trichotillomania across different studies^8,9,26^and a report using data from healthcare services across the US^27^. Also, consistently with previous studies^26^, the most common comorbidities in our cohort were anxiety-related and depressive disorders. Previous studies showed that individuals with both trichotillomania and comorbid anxiety-related or depressive disorders may have more severe hair-pulling symptoms^28^, which may facilitate help-seeking and diagnoses in clinical settings.

We also found relatively high rates of comorbid NDDs and ADHD in particular. To the best of our knowledge, our study is one of the few to report such high rates of clinically-diagnosed comorbid NDDs and ADHD amongst individuals with trichotillomania, adding to a recent study that reported similar rates (29%) of self-reported comorbid ADHD in a cross-sectional sample of adults^12^. One possible explanation for our higher proportion of co-occurring NDDs is that previous trichotillomania studies did not formally assess these disorders, as these assessments are onerous and need to be done by specialists. Additionally, most trichotillomania studies to date have also focused on adults, and it is known that individuals with some diagnoses within the NDDs spectrum (e.g., ADHD, tic disorders) may grow out of their symptoms, or be less impaired by them, in adulthood^29,30^. Also, diagnostic practices have changed over time, with more children (and adults) being diagnosed with NDDs in recent years, which may lead to different estimates across studies over time given the cumulative nature of register-based diagnoses^31,32^. It is also possible that trichotillomania may be more likely to be detected amongst individuals undergoing neuropsychiatric assessments. In any case, these results may have important clinical implications for the management of trichotillomania, as treatments may need to be adapted to suit individuals with NDDs.

Our findings also build upon previous literature on the association between trichotillomania and emotionally unstable (i.e., borderline) personality disorder. Previous studies have reported conflicting findings regarding whether there are higher rates of borderline personality disorder amongst those with trichotillomania^33,34^, or vice versa^35^. Our study identified a higher proportion of borderline personality disorder in individuals with a diagnosis of trichotillomania compared to estimates previously reported in individuals from the general population (1–2%)^36,37^. Trichotillomania may be associated with neuroticism^38,39^and emotion regulation difficulties^40^, and these traits may drive hair-pulling behaviors termed “focused” or “internally-regulated”^41,42^. Additionally, comorbid borderline personality disorder may contribute to non-suicidal self-injury in individuals with trichotillomania^43^and may require treatment adaptations, e.g., the incorporation of elements of dialectical behavior therapy^44^. Given its potential clinical implications, these results indicate that future research should focus on this comorbidity.

Our study also showed a relatively high rate of co-occurring eating disorders among individuals with trichotillomania, which is in line with previous studies in the field, including evidence from a sample of hundreds of individuals with clinically relevant hair-pulling recruited through five centers in two countries^9,26,45^. Individuals with anorexia nervosa, bulimia nervosa, or both commonly have OCD^46^, and evidence from epidemiological and molecular genetic studies indicate shared genetic risk factors between those conditions^47,48^. It is likely that trichotillomania also shares common etiological factors with eating disorders, and future research should examine this question directly.

The TIP^4^was the first study to characterize the pattern of medications used in a large sample of individuals with trichotillomania outside the context of a treatment study. More recently, investigators in the US have also leveraged healthcare records from select services to investigate patterns of dispensed psychotropics in large samples of individuals with trichotillomania^49^. We extend those previous findings by considering nationwide data. We found similar findings that antidepressants (e.g., SSRIs) and anxiolytics were the most frequently dispensed medications. We found higher proportions of dispensed medications than in the TIP study (42%), which may be explained by differences in recruitment and sampling strategies (e.g., not all participants in TIP were treatment-seeking or in specialty services), and we found relatively high rates of dispensed antipsychotics and hypnotics. Overall, the high rates of dispensed medications found in this study are striking, as there are no pharmacological agents with clear evidence of efficacy for trichotillomania^6^. However, the PDR does not include data on the specific indication for a given dispensed medication, and it is likely that, at least to some extent, the dispensed medications were meant to treat the range of co-occurring conditions, rather than (or additionally to) the hair-pulling symptoms. Unfortunately, the registers also do not contain information on psychotherapeutic treatments, even though the evidence for their efficacy is stronger than that for any medication^6^.

The main strengths of our study are the large number of participants with trichotillomania included in the analyses, the nationwide coverage of the Swedish registers, and the quality of the data that were collected. To the best of our knowledge, the largest previous studies reporting on clinical characteristics of individuals with trichotillomania were smaller (n = 858)^22^or relied on self-reported information^4^. However, limitations should also be acknowledged. We did not conduct a formal assessment of validity of the diagnoses (e.g., through evaluation of medical records) as previously done for other conditions^50–52^. However, because the diagnosis of trichotillomania has unique characteristics related to hair-pulling behaviors, misdiagnosis may be less likely and, as discussed above, the sociodemographic and clinical profile of the individuals with trichotillomania in our cohort greatly resemble those described in the literature.

In conclusion, although trichotillomania may be seldom diagnosed by specialists in Sweden, diagnosed individuals in our study had similar sociodemographic and clinical characteristics to those previously reported in the trichotillomania literature. The results open the possibility of register-based research into this underdiagnosed and understudied condition.

## Supplementary Information

Supplementary Information.

## Data Availability

The datasets generated and/or analyzed during the current study are not publicly available due to the Public Access to Information and Secrecy Act in Sweden, which prohibits us from making individual level data publicly available. Researchers who are interested in replicating our work can apply for individual level data to the register holders.
