# Supplementary material for: Sociodemographic and clinical characteristics of 1,234 individuals diagnosed with trichotillomania in the Swedish National Patient Register
Source: Sci Rep. 2025 Mar 26;15:10396. doi: 10.1038/s41598-025-95416-w (PMC11947438; doi:10.1038/s41598-025-95416-w)
Supplement: Supplementary file 1 — Supplementary Information. [file 41598_2025_95416_MOESM1_ESM.pdf]

SUPPLEMENTARY MATERIAL

Sociodemographic and clinical characteristics of 1,234 individuals diagnosed with trichotillomania in the Swedish National Patient Register

Luis C. Farhat, Kayoko Isomura, Lorena Fernández de la Cruz, Anna Sidorchuk, Ralf Kuja-Halkola, Isabell Brikell, Zheng Chang, Brian M. D'Onofrio, Henrik Larsson, Paul Lichtenstein, David Mataix-Cols

Supplementary Table 1. Swedish International Classification of Diseases (ICD), 10th edition codes used to identify diagnoses of psychiatric disorder comorbidities in the Swedish National Patient Register.

**Psychiatric disorders**	**Swedish ICD-10 code**	**Minimal age to retrieve records of diagnoses**
Attention-deficit/ hyperactivity disorder[a]	F90	≥ 3 years
Pervasive developmental disorders	F84	≥ 1 year
Tourette syndrome or chronic tic disorders	F95[b]	≥ 3 years
Schizophrenia or other psychotic disorders	F20, F21, F22, F23, F24, F25 (minus F25.0), F28, F29	≥ 10 years
Bipolar disorders	F25.0, F30, F31, F34.0	≥ 10 years
Depressive disorders (major depressive disorder, persistent mood disorder, and unspecified mood disorder)	F32, F33, F34 (minus F34.0), F38, F39	≥ 6 years
Phobic and anxiety disorders	F40, F41	≥ 6 years
Obsessive-compulsive disorder	F42	≥ 6 years
Reaction to severe stress and adjustment disorders	F43	≥ 6 years
Eating disorders	F50.0-F50.3, F50.9	≥ 8 years
Emotionally unstable personality disorder	F60.3	≥16 years

[a]Individuals with attention-deficit/hyperactivity disorder (ADHD) were also identified by prescription of ADHD drugs, collected from the Prescription Drug Register, specifically amphetamine (Anatomical Therapeutic Chemical [ATC] Classification System code: N06BA01), dexamphetamine (N06BA02), methylphenidate (N06BA04), atomoxetine (N06BA09), and lisdexamphetamine (N06BA12).

[b]Tourette syndrome and chronic tic disorder cases were ascertained as based on the algorithm described in Rück et al. (2015).

Supplementary Table 2. Swedish Anatomical Therapeutic Chemical (ATC) Classification System codes used to identify dispensed medications in the Swedish Prescribed Drug Register.

	ATC codes
**ADHD medications**	N06BA
Methylphenidate	N06BA04
Other ADHD medications	N06BA01, N06BA02, N06BA09, N06BA12
**Antidepressants**	N06A
SSRIs	N06AB
Clomipramine	N06AA04
Other antidepressants	N06A (excluding N06AA04 and N06AB)
**Anxiolytics**	N05B
Benzodiazepine-based	N05BA
Non-benzodiazepine-based	N05BB (excluding N06BA)
**Hypnotic/ sedatives**	N05C
Benzodiazepine-based	N05CD, N05CF
Non-benzodiazepine-based	N05C (excluding N05CD and N05CF)
**Antipsychotics**	N05A
Typical antipsychotics	N05AA, N05AB, N05AC, N05AD, N05AF, N05AG, N05AK, N05AL
Atypical antipsychotics	N05AE, N05AH, N05AX
**Lithium**	N05AN
**Antiepileptics**	N03A
Lamotrigine	N03AX09
Valproic acid	N03AG01
Other antiepileptics	N03A (excluding N03AG01 and N03AX09)
**Analgesics**	N02A, N02BF
**Antiparkinsonian medications**	N04
**Naltrexone**	N07BB04
**Analgesics**	N02
Opioids	N02A
Other analgesics and antipyretics	N02B
Antimigraine preparations	N02C
**Other nervous system medications**	N07 (excluding N07BB04)

Abbreviations: ADHD, attention-deficit/hyperactivity disorder; SSRI, selective serotonin reuptake inhibitors.
